# Tumours and tremors: how PTEN regulation underlies both

**DOI:** 10.1038/sj.bjc.6602994

**Published:** 2006-02-21

**Authors:** R H Kim, T W Mak

**Affiliations:** 1 Campbell Family Institute for Breast Cancer Research, Toronto, Ontario M5G 2C1, Canada; 2 Ontario Cancer Institute, Toronto, Ontario M5G 2C1, Canada; 3 Department of Medical Biophysics, University of Toronto, Toronto, Ontario M5G 2C1, Canada

**Keywords:** PTEN, DJ-1, tumour suppression, Parkinson's disease

## Abstract

Mutations of the tumour suppressor PTEN (phosphatase and tensin homolog deleted on chromosome 10) are seen in many human cancers. However, dysregulation of PTEN may be involved in other disease states such as Parkinson's disease. This minireview describes recent work examining PTEN regulation and its implications for the development of both cancer and neurodegenerative disease.

## PTEN AND TUMORIGENESIS

Almost a decade has passed since the discovery of the lipid phosphatase PTEN (phosphatase and tensin homolog deleted on chromosome 10) as an important tumour suppressor. The *PTEN* gene was originally identified by three independent groups using different methodologies: a representational difference analysis of tumours; a high-density scan of chromosome 10q in glioma cell lines and a screening of human cDNA libraries designed to detect protein phosphatases upregulated in response to transforming growth factor *β* (TGF-*β*) (Maehama *et al*, 2001). Mutations and deletions in the *PTEN* locus were subsequently found to be associated with a broad range of human cancers. While mutations occur throughout the length of the *PTEN* gene, tumorigenic alterations are clustered in exon 5 encoding the catalytic domain, confirming that the PTEN lipid phosphatase activity is critical for tumour suppression (Ali *et al*, 1999).

Loss of heterozygosity (LOH) studies comparing early and advanced cancers suggest that PTEN may play its most important role in advanced cancers of particular tissues (Ali *et al*, 1999). For example, the mutation frequency of *PTEN* is high in malignant glial tumours, but much more modest in lower grade gliomas and glioneuronal tumours. Furthermore, alterations of *PTEN* in glial tumours are associated with shorter median survival. Prostate tumours are associated with LOH of 10q23, with mutations of *PTEN* reported most frequently in metastatic disease and in cases with a poor prognosis. Although second mutations of *PTEN* occur less frequently than LOH, loss of PTEN protein occurs frequently in high grade and Gleason score prostate tumours. *PTEN* is also considered as the most frequently mutated gene in endometrial cancers. Indeed, mutation of *PTEN* likely contributes to the initiation of endometrial tumorigenesis, as this gene is also altered in premalignant endometrial hyperplasia (Sansal and Sellers, 2004).

Germline mutations in tumour suppressor genes are associated with cancer predisposition syndromes that result in the appearance of hamartomas (hyperplastic and disorganised growths) throughout the body. Germline mutations of the *PTEN* gene are found in two hamartoma disorders called Cowden syndrome (MIM 158350) and Bannayan–Riley–Ruvalcaba (MIM 153480) syndrome. Cowden syndrome is a rare, autosomal dominant, familial cancer syndrome characterised by hamartomas, multiple smooth facial papules, acral keratosis and multiple oral papillomas. In contrast, Bannayan–Riley–Ruvalcaba syndrome is marked by macrocephaly, lipomatosis, hemangiomatosis and speckled penis (Eng, 2003). As with sporadic tumours, the germline *PTEN* mutations associated with these hamartoma syndromes are clustered around the catalytic site (Ali *et al*, 1999).

Enhanced tumorigenesis has also been observed in Pten-deficient mice. Numerous conventional and conditional gene-targeting murine models of *pten* deficiency have been generated to investigate the physiological functions of PTEN. Conventional gene-targeting of *pten* in all mouse tissues (*pten*^−/−^ mice) results in developmental delay and lethality at embryonic days 6.5–8.5 due to a failure in chorio-allantoic fusion (Kishimoto *et al*, 2003). However, *pten*^+/−^ mice eventually develop LOH of the remaining *pten* allele, leading to the appearance of tumours in the endometrium, liver, prostate, gastrointestinal tract, thyroid and thymus. Interestingly, in addition to tumours, tissue-specific deletion of *pten* can result in hyperplasia, autoimmunity, glucose dysregulation or neurological deficits (Kishimoto *et al*, 2003).

## STRUCTURE OF THE *PTEN* GENE

The human genomic *PTEN* locus consists of nine exons encoding a 5.5 kb mRNA that specifies a 403 amino-acid open reading frame. The translation product is a 53 kDa protein with extensive homology to tensin/auxilin and protein tyrosine phosphatases (PTPs). Studies of the crystal structure of PTEN have revealed an enlarged catalytic site (relative to other PTPs) that accommodates large phosphoinositide substrates, and a C2 domain that mediates the recruitment of proteins to the membrane (Maehama *et al*, 2001). In addition to the C2 domain, the C-terminus of PTEN contains a PSD95/SAP90, DLG, ZO-1 (PDZ)-binding domain (involved in protein–protein interactions) and two proline, glutamic acid, serine and threonine (PEST) sequences (involved in protein degradation) (Leslie and Downes, 2004).

## FUNCTIONS OF THE PTEN PROTEIN

The N-terminus of the PTEN protein is similar to that of PTPs, and PTEN was initially thought to be a dual specificity PTP. Indeed, the PTP activity of PTEN has been implicated in cellular processes such as cell motility (Raftopoulou *et al*, 2004). However, PTEN removes phosphate groups from phosphorylated threonine and serine protein targets only very inefficiently, and its lipid phosphatase activity is now considered its most important attribute. PTEN is most active against negatively charged peptides, and its primary cellular substrate is the membrane lipid phosphoinositide-3,4,5-trisphosphate (PIP_3_) (Maehama *et al*, 2001). Phosphatidylinositol 3,4,5-trisphosphate is the principal second messenger of the phosphoinositide-3 kinase (PI3′K) pathway that mediates receptor tyrosine kinase signalling through to the survival kinase PKB/Akt (Chan *et al*, 1999). Increased levels of PIP_3_ at the membrane cause PH domain-containing proteins such as PKB/Akt and PDK-1 to colocalise, resulting in PDK-1-mediated phosphorylation and activation of PKB/Akt. Activated PKB/Akt transfers a phosphate group to target proteins involved in cell survival, cell cycling and metabolism (Chan *et al*, 1999). PTEN exerts its tumour-suppressive effect by dephosphorylating PIP_3_, thereby negatively regulating PKB/Akt activation and the PI3′K survival pathway (Maehama *et al*, 2001).

PTEN-mediated modulation of PI3′K signalling plays a key role in regulating cellular functions associated with proliferation/cell cycle, programmed cell death (PCD), angiogenesis and migration. Engineered overexpression of PTEN induces profound growth suppression primarily by promoting cell cycle arrest. For example, PTEN overexpression leads to G1 arrest in glioma, breast, endometrial and prostate cancer cells (Maehama *et al*, 2001). This cell cycle arrest requires PTEN's lipid phosphatase activity, can be enhanced by low serum, and can be rescued with the introduction of constitutively active forms of PI3′K, PKB/Akt or PDK-1 (Maehama *et al*, 2001). One mechanism by which PTEN induces cell cycle arrest is by regulating PKB/Akt such that levels of the cell cycle inhibitor p27^kip1^ are increased. Overexpression of PTEN also correlates with decreased total levels and nuclear localisation of cyclin D1, a key cell cycle molecule regulated by PKB/Akt. Other PKB/Akt-regulated cell cycle mediators affected by PTEN expression levels include the forkhead transcription factors and glycogen synthase kinase (Maehama *et al*, 2001).

In addition to regulating the cell cycle, PTEN controls various forms of PCD. Overexpression of PTEN induces the apoptosis of many different cell types, and *pten*-deficient murine embryonic fibroblasts (MEFs) are resistant to various apoptotic stimuli (Maehama *et al*, 2001). PTEN's promotion of the apoptotic response may cooperate with PTEN's function in cell cycle arrest, and may be mediated by more than one mechanism. Through its effects on PKB/Akt, PTEN can induce the activation of proapoptotic molecules such as Fas and bim, while promoting the inactivation of antiapoptotic molecules such as the bcl-2 family member Bad and the X-linked inhibitor of apoptosis (XIAP) that blocks caspase activation. As a result, *pten*^+/−^ mice are abnormally susceptible to Fas-mediated apoptosis, and ectopic expression of PTEN sensitises glioblastoma cells to irradiation- and Fas-induced apoptosis characterised by increased caspase-3 activity (Kishimoto *et al*, 2003). PTEN also plays an important role in the induction of death in cells that lose contact with the extracellular matrix, a type of PCD called anoikis. Reconstitution of PTEN in *PTEN*-deficient cells restores anoikis by negatively regulating the scaffold protein focal adhesion kinase (FAK). A detectable decrease in FAK phosphorylation is also observed when PTEN overexpression results in inhibition of fibronectin-induced formation of actin stress fibres and prevention of cell spreading, migration on extracellular matrix proteins and invasion. Conversely, an absence of PTEN function may allow unregulated cell spreading and invasion that could contribute to metastasis (Maehama *et al*, 2001).

In addition to promoting the survival and metastasis of tumour cells, *PTEN* mutations can contribute to malignancy through altered regulation of protein synthesis and cellular nutrient consumption. Through studies using the fruitfly *Drosophila melanogaster*, it has been shown that PTEN inhibits the target of rapamycin (mTOR) metabolic pathway. Dysregulation of this pathway resulted in altered protein translation and amino-acid consumption, which in turn led to marked increases in individual cell size and organ size. As well, recent studies have suggested that *PTEN* mutations can influence the activity of the vascular endothelial-like growth factor (VEGF) that stimulates blood vessel formation. Mutation of *PTEN* can destabilise hypoxia-inducible transcription factor 1 (HIF-1), the molecule that drives *VEGF* transcription. Thus, in the absence of PTEN, a combination of increased metabolism and enhanced angiogenesis may promote tumour cell growth and metastasis (Sansal and Sellers, 2004).

## REGULATION OF THE PTEN PROTEIN

Some tumours, such as sporadic breast cancers, contain wild-type *PTEN* genes but decreased PTEN protein immunoreactivity. This observation implies that dysregulation of a normal *PTEN* gene or protein can abrogate PTEN function and lead to neoplastic disease. *PTEN* was initially cloned as a gene whose transcription was downregulated by TGF-*β*. However, in addition to transcriptional regulation, the function of the normal PTEN protein can be modulated by protein–protein interactions, phosphorylation and other epigenetic factors.

The C-terminus of PTEN is critical for its tumour suppressive activity and contains putative phosphorylation sites thought to play a role in PTEN regulation (Leslie and Downes, 2004). Residues Ser380, Thr382 and Thr383 are substrates for phosphorylation by casein kinase II (CK2). Casein kinase II-mediated phosphorylation of these residues alters electrostatic shielding and decreases the affinity of the catalytic and C2 domains of PTEN for the membrane, decreasing PTEN activity. Casein kinase II-mediated phosphorylation also stabilises the PTEN protein by preventing its proteasomal degradation and proteolysis by caspases, while keeping it in an inactive state. Inhibition of CK2-mediated PTEN phosphorylation results in increased PTEN activity and a corresponding reduction in PKB/Akt activation. This activation of unphosphorylated PTEN may be due to a conformational change that opens the PDZ-binding sites. The presence of open PDZ-binding sites in PTEN allows interactions with PDZ-containing proteins such as membrane-associated guanylate kinase inverted-2 (MAGI-2), as well as the formation of active PTEN-associated complexes that can decrease PKB/Akt activation (Leslie and Downes, 2004). Overexpression of MAGI-2 has been shown to restore PTEN stability in vinculin null F9 cells, which are cells that contain decreased PTEN protein but normal levels of *PTEN* mRNA (Subauste *et al*, 2005). Other PDZ domain-containing proteins that interact with PTEN include MAGI-3, hDLG (discs-large) and hMAST (microtubule-associated serine-threonine kinase), all of which can bind to PTEN and affect its stability through phosphorylation of the C-terminal tail (Leslie and Downes, 2004; Valiente *et al*, 2005).

Another PTEN-binding protein, thioredoxin, is a redox protein that is overexpressed in a large number of tumours. Cys32 of thioredoxin forms a disulphide bond with Cys212 in the C2 domain of PTEN, inhibiting PTEN activity (Meuillet *et al*, 2004). PTEN can also be reversibly inactivated by exposure to hydrogen peroxide, which induces the formation of an internal disulphide bond between Cys71 and Cys124 in the catalytic core (Lee *et al*, 2002). This type of PTEN inactivation is seen in cell cultures following growth factor-induced peroxide production, suggesting that redox inactivation of PTEN is a physiologic response to mitogen stimulation (Kwon *et al*, 2004). Consistent with this hypothesis, increases in hydrogen peroxide specifically induced by mitochondrial dysfunction lead to the oxidation and inactivation of PTEN and increased PI3′K signalling (Connor *et al*, 2005). The ability of oxidised PTEN to regain its tumour suppressive activity depends on the capacity of thioredoxin to reduce the inactive form, and a total loss of PTEN function can occur if the redox status of the cell is abnormal (Lee *et al*, 2002; Kwon *et al*, 2004).

At the mRNA level, analyses of various tumours have shown that methylation of the *PTEN* promoter can result in transcriptional silencing of the *PTEN* gene (Kang *et al*, 2002). Interestingly, the function of the *PTEN* promoter and PTEN mRNA expression appear to be regulated by more than one transcription factor. For example, the genomic *PTEN* promoter sequence contains a GC-rich 5′untranslated region that can be activated by the transcription factor Sp1 to drive constitutive PTEN expression (Han *et al*, 2003). Another transcription factor, early growth response-1 (Egr-1), binds to the *PTEN* untranslated region and activates *PTEN* transcription during irradiation-induced signalling. The *PTEN* promoter also contains binding sites for another irradiation-sensitive transcription factor, p53, which has been shown to increase levels of both PTEN mRNA and protein (Leslie and Downes, 2004). NF-*κ*B increases PTEN expression during cellular differentiation but decreases *PTEN* transcription in cells responding to apoptotic stimuli, suggesting that NF-*κ*B-induced transcription of *PTEN* may depend on the cellular context. Finally, a ligand-activated nuclear receptor called peroxisome proliferator-activated receptor (PPAR*γ*) is involved in anti-inflammatory responses thought to be mediated by transcriptionally controlled upregulation of PTEN (Leslie and Downes, 2004).

## USE OF *D. MELANOGASTER* TO INVESTIGATE PTEN SIGNALLING

The PI3′K signalling pathway regulated by PTEN is largely conserved in metazoans, and PTEN and PI3′K homologues have been identified in many species. In addition to its effect on translation, much has been learned about the biological functions of PTEN and the PI3′K pathway from the fruit fly, *D. melanogaster. Drosophila* PI3′K signalling involves homologues of insulin receptor ligand (CHICO), the insulin/IGF-1 receptor (DInr), PI3′K (Dp110), PTEN (dPTEN) and PKB/Akt (dPKB/dAkt1) (Simpson and Parsons, 2001). Mutation of any one of the genes encoding these proteins produces an activation of PI3′K signalling that drives cellular expansion and proliferation and leads to increases in cell size, cell number and organ size. Conversely, overexpression of dPTEN or dominant-negative dPI3′K in *Drosophila* embryos triggers apoptosis similar to that observed in dPKB/dAkt1- or dPDK1-deficient flies. In the *Drosophila* eye, PTEN overexpression inhibits cell cycle progression and promotes cell death (Maehama *et al*, 2001). In our own work, we used a system based on this latter observation to isolate dPTEN regulators in a gain-of-function screening protocol employing a P-element library. We identified *DJ-1*, a putative oncogene involved in autosomal recessive early-onset Parkinson's disease (PD) (Bonifati *et al*, 2003), as a novel suppressor of PTEN function (Kim *et al*, 2005a).

## LINKS BETWEEN DJ-1 AND TUMORIGENESIS

Several lines of evidence suggest that DJ-1 plays a role in human tumorigenesis. Breast cancer patients have elevated levels of serum DJ-1 and circulating anti-DJ-1 autoantibodies compared to healthy and non-breast cancer patients (Le Naour *et al*, 2001). Furthermore, DJ-1 protein is increased in primary non-small-cell lung carcinoma samples (MacKeigan *et al*, 2003). As with prostate cancer, the rate of LOH at 10q23 in the lung and breast cancers is greater than the rate of mutation of the remaining *PTEN* allele. This observation suggests that, in these cases, epigenetic regulation of *PTEN* has caused its loss of function. Although the underlying mechanism has yet to be clearly defined, it is quite possible that faulty control of a PTEN regulator such as DJ-1 is responsible. In lung cancer patients, an elevation in *DJ-1* transcripts correlates with a poor prognosis, particularly for Stage I disease (Kim *et al*, 2005a). Higher levels of *DJ-1* mRNA are also present in patients lacking mutations in Ras, a well-known molecular marker of tumorigenesis. These data suggest that DJ-1 is important for tumour initiation and may be a useful prognostic marker in certain types of lung cancer. In primary breast cancer samples, DJ-1 expression correlates positively with phospho-PKB/Akt immunoreactivity. *In vitro*, DJ-1 inhibits PTEN's negative regulation of the PI3′K pathway and increases PKB/Akt activation. Thus, DJ-1 is likely oncogenic because of its negative regulatory effects on PTEN, effects that indirectly promote activation of the PI3′K cell survival pathway.

## LINKS BETWEEN PTEN, DJ-1 AND PD

Parkinson's disease and cancer are two pathologic processes resulting from excessive signalling by one of two sets of opposing forces: those driving cell death and those promoting cell survival. Parkinson's disease results from the excessive death of dopaminergic neurons in the *substantia nigra pars compacta* (SNpc) in the brain, while tumorigenesis is driven by excessive cell survival in the target tissue. The underlying signalling mechanisms of cell death and survival can be generalised and applied to most cell types and tissues, implying that the balance can be shifted from death to survival and *vice versa* by aberrations of the same signalling pathway. Early epidemiological studies showed a decreased incidence of cancer in PD patients, later confirmed in larger studies (West *et al*, 2005). Moreover, the genes associated with familial forms of PD (the PARK loci) are now implicated in tumorigenesis. For example, *α*-synuclein (*SNCA*; PARK1) expression is upregulated in glioma cell lines, schwannomas, medulloblastomas and breast and ovarian carcinomas. Deletions of *Parkin* (PARK2) have been identified in hepatocellular carcinomas and in breast, ovarian and non-small-cell lung cancers. Finally, ubiquitin C-terminal hydrolase (*UCH-L1*; PARK5) is overexpressed in oesophageal and squamous cell carcinomas, and in pancreatic and colorectal cancers (West *et al*, 2005). The effects of PARK mutations on tumorigenesis are unclear and, due to the unknown functions of these PD-associated genes, conclusions about the nature of these mutations (gain-of-function *vs* loss-of-function) are difficult to establish. Moreover, loss/gain-of-function mutations in PD may have additional distinct effects on tumorigenesis, confounding attempts to ‘pigeon-hole’, a particular gene. Nevertheless, genes associated with familial PD have been shown to regulate cell death and/or the cell cycle, and several lines of evidence imply that malfunction of a shared biochemical pathway may lead to PD or cancer. Firstly, during tumorigenesis, the degradation of p27^kip1^ can be driven by either PTEN or UCH-L1 (Caballero *et al*, 2002; Viglietto *et al*, 2002). Secondly, deletions of the *Parkin* gene in *Drosophila* result in PKB/Akt activation (Yang *et al*, 2005). Thirdly, *PINK1*, which encodes a kinase downregulated in the absence of PTEN, has been identified as the sixth locus (PARK6) associated with familial PD (West *et al*, 2005).

A key link between PTEN dysregulation and PD may be DJ-1, isolated originally as PARK7. The identification of loss-of-function mutations in the *DJ-1* gene in PARK7 PD families is consistent with the notion that DJ-1 acts to promote neuron survival via the PI3′K signalling pathway. The biochemistry of the neurodegeneration in PD points to mitochondrial oxidative stress as the mechanism driving neuronal death in the SNpc. In *Drosophila*, an siRNA-induced reduction of DJ-1 causes neurons to become hypersensitive to oxidative stress, and these flies show neurodegeneration that is exacerbated by coexpression of PTEN (Yang *et al*, 2005). Significantly, this hypersensitivity to oxidative stress is also seen in the neurons of *dj-1*-deficient mice (Kim *et al*, 2005b). In both organisms, the increased susceptibility to oxidative stress can be rescued by activation of the PI3′K pathway (Kim *et al*, 2005b; Meulener *et al*, 2005; Yang *et al*, 2005). Other work has implicated DJ-1 as a sensor of reactive oxygen species and as a molecular chaperone (Shendelman *et al*, 2004), and DJ-1 may be able to influence the protein stability or oxidative state of elements of the PI3′K pathway. For example, PTEN and ASK1, a component of a DJ-1-containing complex (Junn *et al*, 2005), are both regulated by thioredoxin (Meuillet *et al*, 2004). These findings suggest that DJ-1 may act in cooperation with redox-sensitive proteins to regulate its substrates, and that DJ-1-mediated regulation of one of these substrates, PTEN, plays a vital role in preventing both cancer and neurodegenerative disease.

## CONCLUSIONS AND PERSPECTIVES

The tumour suppressor gene *PTEN* is currently recognised as one of the most frequently mutated genes in human cancers. As befits a critical gene, PTEN is regulated at multiple levels, including transcription, protein stability and phosphorylation. Understanding this regulation is crucial for the effective design of novel cancer therapeutics. Inhibitors of the PI3′K pathway such as wortmannin and LY294002 are commonly employed in the research laboratory, while receptor tyrosine kinase inhibitors such as gefitinib (Iressa; AstraZeneca, Wilmington, DE, USA), trastuzumab (Herceptin; Genentech, San Francisco, CA, USA) and imatinib (Gleevec; Novartis, Summit, NJ, USA) have been added to the clinical armamentarium used to treat certain types of human cancers. Inhibitors of downstream effectors of the PI3′K pathway, such as the rapamycin derivatives CCI-779 (Wyeth Research, Madison, NJ, USA) and RAD001 (Novartis) are under investigation to determine their efficacy as mTOR inhibitors in tumours. Similarly, because oxidative stress and reactive oxygen species can inactivate PTEN, alternative therapeutics that target PTEN's redox status are in the development phase.

The involvement of the PI3′K pathway and putative regulators of PTEN (such as DJ-1) in PD indicates that the importance of PTEN regulation extends beyond the known roles of this gene in tumorigenesis, hamartomatous syndromes and autoimmunity. As PTEN is regulated by phosphorylation and degradation via the ubiquitin–proteasome system, proteasomal targeting may offer a potential therapeutic modality for the treatment of PTEN-related cancers. For example, the proteasome inhibitor bortezomib (Velcade; Millennium Pharmaceuticals, Cambridge, MA, USA) has been effective in treating multiple myeloma because survival factors that depend on proteasomal degradation (such as NF-êB) are inactivated. However, caution is warranted in using this approach, as other proteasome inhibitors induce Parkinsonian-like symptoms when administered to rats (McNaught *et al*, 2004). Intriguingly, this latter finding offers additional support for the hypothesis that cancer and PD may both result from PTEN dysregulation. All these developments point towards exciting new avenues for the complete elucidation of PTEN's physiological functions, and the manipulation of this knowledge for the treatment of both tumorigenesis and neurodegeneration.
